# Supplementary material for: simATAC: a single-cell ATAC-seq simulation framework
Source: Genome Biol. 2021 Mar 4;22:74. doi: 10.1186/s13059-021-02270-w (PMC7934446; doi:10.1186/s13059-021-02270-w)
Supplement: Supplementary file 4 — Additional file 4 Review history. [file 13059_2021_2270_MOESM4_ESM.docx]

Review History

**First round of review**

**Reviewer 1**

**Were you able to assess all statistics in the manuscript, including the appropriateness of statistical tests used?**

Yes: Statistical tests were described in Results and Method. Codes to reproduce their results are open-accessible online.

**Were you able to directly test the methods?**

Yes

**Comments to author:**

In this work, Ghaziani, Zhang and Wang presented a novel method to simulate single-cell ATAC-seq data from real scATAC-seq data. The tool can simulate scATAC-seq by modeling the library size, mean counts of bins, and non-zero cell proportions of bins from real scATAC-seq data, and authors showed that the simulated data mimic the real data. The software tool is well made with detailed documentation, flexible parameters, and satisfying efficiency to finish simulation in minutes. This simulation method will provide a benchmarking framework for the field to develop and evaluate new methods for single-cell ATAC-seq analysis. However I have the following major and minor comments:

1. In the Result section of "Simulating feature matrices", authors claimed that simATAC is able to generate peak-by-cell count matrix which is commonly used to represent scATAC-seq data. However, according to the simATAC code (https://github.com/bowang-lab/simATAC/blob/master/R/ExtraFunctions.R), such "simATACgetCellByPeak" function is in fact to generate a "peak-bin"-by-cell count matrix, or in another word, a subset (the top X bins by mean value) of the default output from simATAC -- the bin-by-cell count matrix. Such representation is far different with the actual peak-by-cell count matrix people usually use, where peak should have variable length instead of a fixed length of e.g. 5kb. Authors showed that in Table 4, peak regions, top 5000 real bins and top 5000 simulated bins are overlapping. However, there is no evidence to show that the downstream analysis on the simATAC "peak-bin"-by-cell matrices will be close to real data on the peak-by-cell matrices, as what authors showed in Table 5 on analyses based on bin-by-cell matrices. Also, it's not clear if the Table 4 result is robust and how many simulation runs had been done to generate Table 4 result.

2. In Table 5, what does the 'Real' row mean? I assume that 'Real' means the 'real' noise level in the real data. If so, does the result show that with the same noise level as the real data, the simulated data yielded the worst NMI, AMI and ARI? Overall, the NMI, AIM, and ARI shows big difference between simulated data and real data especially in Buenrostro2018 and PBMCs datasets, considering the same clustering analysis (snapATAC) was used. It seems that the cell specific accessibilities were not maintained well even with noise level 0. By the way, please explain how NMI, AMI, and ARI are calculated in the Method or provide the references, as what authors explained for MAD, MAE and RMSE.

3. In the Method section of "simATAC statistical modelling", authors claimed that they "observed a significant difference in the distribution of library sizes between the real scATAC-seq data generated by the 10xG platform and other platforms", so that they use unimodal Gaussian model to fit 10xG sample and bimodal GMM for samples from other platform. Please justify such observation at least in a supplementary material. Also, please write the whole name of "10x Genomics" when first using the abbreviation "10xG".

4. Software dependencies. `simATAC` depends on SnapTools which is further depending on Python 2.X and R version 3. However, the Python 2.X has been obsolete after Dec of 2019, the current active R is version 4, and there are quite a lot of changes between Python2 and Python3, between R 3.X and R 4.X. Please make sure this new tool can work on the currently active Python and R environment.

5. Please discuss the potential usage of the simulated single-cell ATAC-seq data while evaluating computational tools. Authors showed that simATAC can mimic real data. However, in reality, why users should use these simulated data to evaluate their tools instead of simply testing their tools on real data which the simulation is based on?

Minor:

1. Please double-check if the description at the end of Result section "Bin by cell count matrix" on the "SCESet" object is right. According to simATAC tutorial at https://github.com/bowang-lab/simATAC/blob/master/Docs/tutorial.md, it should be just called as "SCE" object.

**Reviewer 2**

**Were you able to assess all statistics in the manuscript, including the appropriateness of statistical tests used?**

No

**Were you able to directly test the methods?**

No

**Comments to author:**

The manuscript "simATAC: A Single-cell ATAC-seq Simulation Framework" by authors Ghaziani et al., presents a new tool to create synthetic single cell ATAC-seq (scATAC-seq) data derived from experimentally generated scATAC-seq characteristics. Unlike other tools, simATAC segments the genome into equally-sized bins before simulating data based on three parameters: library size, sparsity, and average number of reads per bin. simATAC also contains a feature to control the amount of simulated noise in the synthetic data set. The authors describe simATAC as a means to generate synthetic scATAC-seq data for software benchmarking, but it is likely to be useful in other instances where simulated data is required.

One of the biggest advantages of simATAC is that it is tested against data generated from three of the most common scATAC-seq technologies. Technology significantly influences the quality and characteristics of scATAC-seq data, but simATAC simulates data that is consistently similar to real data regardless of the platform. The authors also demonstrate how adjusting the noise parameter influences the clustering analyses. Given the impact of the signal-to-noise ratio on peak detection, this is an important factor when benchmarking peak-calling software. And perhaps most important from a user's perspective, the simATAC software is well documented and its tutorial is thorough.

There are a few points that need to addressed in the manuscript. Firstly, in Figure 3 and several supplemental figures, the cell sparsity of simulated data is consistently lower than that of real data. It would be helpful to simATAC users if the authors addressed this. In these same figures the authors plot bin sparsity of real and simulated data. Due to the nature of this metric, the majority of data points cluster between 0.75 and 1.0, making it difficult to determine if there are differences in median and quartile distributions as is observed with the cell sparsity plots. It would be useful if the authors plotted these differently to increase the resolution in the bin sparsity plots.

Secondly, Table 3 compares the similarity between real and synthetic data using three metrics (MAD, MAE, RMSE), and presents these data as an average of all cell groups. Since scATAC-seq data varies significantly based on individual cell type, it would be useful describe the range of variation across the tested cell groups.

Finally, Table 4 shows peak assignment similarities in simulated and real data. However, the authors only use the peak caller SnapATAC. Like simATAC, SnapATAC segments the genome into 5kb bins. To robustly demonstrate the similarity between simulated and real data sets the authors should also apply a non-segmenting peak caller. Because of the variety in peak calling software, it would be useful to compare real and simulated data using different approaches.

simATAC is an important tool in the field of data simulation. While many current approaches use subsetting of experimental data for benchmarking, simATAC provides a new way to generate completely synthetic data set based on real data parameters.

**Authors Response**

**Point-by-point responses to the reviewers’ comments:**

Response to Reviewer #1:

Response to the main concern:

[[...]]

In this work, Ghaziani, Zhang and Wang presented a novel method to simulate single-cell ATAC-seq data from real scATAC-seq data. The tool can simulate scATAC-seq by modeling the library size, mean counts of bins, and non-zero cell proportions of bins from real scATAC-seq data, and authors showed that the simulated data mimic the real data. The software tool is well made with detailed documentation, flexible parameters, and satisfying efficiency to finish simulation in minutes. This simulation method will provide a benchmarking framework for the field to develop and evaluate new methods for single-cell ATAC-seq analysis.

However I have the following major and minor comments:

Major comments:

1. In the Result section of “Simulating feature matrices”, authors claimed that simATAC is able to generate peak-by-cell count matrix which is commonly used to represent scATAC-seq data. However, according to the simATAC code (https://github.com/bowang-lab/simATAC/blob/master/R/ExtraFunctions.R), such “simATACgetCellByPeak” function is in fact to generate a “peak-bin”-by-cell count matrix, or in another word, a subset (the top X bins by mean value) of the default output from simATAC -- the bin-by-cell count matrix. Such representation is far different from the actual peak-by-cell count matrix people usually use, where peaks should have variable length instead of a fixed length of e.g. 5kb. Authors showed that in Table 4, peak regions, top 5000 real bins and top 5000 simulated bins are overlapping. However, there is no evidence to show that the downstream analysis on the simATAC "peak-bin"-by-cell matrices will be close to real data on the peak-by-cell matrices, as what authors showed in Table 5 on analyses based on bin-by-cell matrices. Also, it's not clear if the Table 4 result is robust and how many simulation runs had been done to generate Table 4 results.

*We thank the reviewer for these comments. The main functionality of simATAC is to simulate bin-by-cell scATAC-seq count matrices. However, it also provides the users additional features such as converting the simulated bin-by-cell matrices to peak-by-cell feature matrices by filtering bins enriched in aligned reads, and this can be achieved using the “simATACgetCellByPeak” function. We clarified the definition of peak-by-cell matrix extracted by simATAC in the “Simulating feature matrices” section.*

*To demonstrate the resemblance of the simulated peak-by-cell matrices to input real data, we took reviewer's suggestion and performed downstream analysis. We extracted peak-by-cell matrices from the bin-by-cell matrices using the three benchmarking datasets as input. We then used Seurat to perform clustering analysis on the simulated peak-by-cell matrices and compared the results with those of real datasets (peak-by-cell matrices extracted from input bin-by-cell matrix), provided in Additional File 1 Figure S8. Note that we use Seurat instead of SnapATAC because SnapATAC is designed for analyzing bin-by-cell matrices. The solid lines in Additional File 1 Figure S8 represents the clustering metrics (NMI, AMI, ARI) of the simulated peak-by-cell matrices, varying the number of peak bins (30000, 40000, 50000 and 60000) and Gaussian noise levels (mean: 0, std: 0; mean: -0.3, std: 0.3; mean: -0.4, std: 0.4; mean: -0.5, std: 0.5). The dotted lines represent the clustering metrics of the extracted peak-by-cell from real bin-by-cell matrices. Each reported metric in Additional File 1 Figure S8 is averaged over 20 simulation runs, which demonstrates the resemblance of the simulated peak-by-cell matrices to the real data as well as its robustness. The results of our new analyses show that the extracted peak-by-cell matrices based on simATAC simulated data maintain the cell-specific accessibility information of the real input data, and is robust to downstream analyses.*

2. In Table 5, what does the 'Real' row mean? I assume that 'Real' means the 'real' noise level in the real data. If so, does the result show that with the same noise level as the real data, the simulated data yielded the worst NMI, AMI and ARI? Overall, the NMI, AIM, and ARI shows big differences between simulated data and real data especially in Buenrostro2018 and PBMCs datasets, considering the same clustering analysis (snapATAC) was used. It seems that the cell specific accessibilities were not maintained well even with noise level 0.

By the way, please explain how NMI, AMI, and ARI are calculated in the Method or provide the references, as what authors explained for MAD, MAE and RMSE.

*We thank the reviewer for pointing out this confusion and we have clarified it in the revised manuscript. "Real" in previous Table 5 (current Table 4) represents the clustering results using the real scATAC-seq data (bin-by-cell matrix), and there is no simulation involved in this evaluation. While the other rows are the clustering metrics using the simulated bin-by-cell matrices generated by simATAC, with noise levels (no noise; mean: -0.3, std: 0.3; mean: -0.4, std: 0.4). Note that the adding Gaussian noise procedure of simATAC is not simply adding noises to the input real data. Instead, simATAC firsts simulates a synthetic bin-by-cell matrix based on multiple statistical models, and Gaussian noises are added to this matrix to represent the high noise and sparsity nature of real scATAC-seq.*

*To clarify this, we include a detailed explanation in the caption of the new Table 4. All the clustering result metrics were reported based on the Louvain clustering algorithm of SnapATAC, as well as simulated bin-by-cell matrices generated by simATAC with different levels of noise (mean: 0 and std: 0 (no Gaussian noise included), mean: -0.3 and std: 0.3; mean: -0.4 and std: 0.4).*

*In the revised manuscript, we added the definitions of NMI, AMI and ARI to the Evaluation metrics section under the Method section, and a more detailed description to Additional File Note S2.*

3.	In the Method section of "simATAC statistical modelling", authors claimed that they "observed a significant difference in the distribution of library sizes between the real scATAC-seq data generated by the 10xG platform and other platforms", so that they use unimodal Gaussian model to fit 10xG sample and bimodal GMM for samples from other platform. Please justify such observation at least in supplementary material. Also, please write the whole name of "10x Genomics" when first using the abbreviation "10xG".

*We thank the reviewer for this comment. We conducted the Kolmogorov-Smirnov test, the goodness of fit Chi-squared test and Hartigan's dip test in all samples to check whether the log-transformed library sizes follow a univariate Gaussian distribution. Most of the samples sequenced on the 10x Genomics Chromium (10xG) reject the null hypothesis at significance level = 0.05, while most of the non-10xG samples fail to reject the null hypothesis. The results are provided in Additional File 3.*

Further, we conducted hypothesis testing to check if the 10xG log-transformed library sizes follow a bivariate Gaussian distribution, and most of the samples fail to reject the tests at = 0.05. Thus, we model the log-transformed library sizes using a bivariate Gaussian distribution if the sample is sequenced on 10xG platform, and we model those of non-10xG samples using a univariate Gaussian distribution (which can be seen as a special case of a bivariate Gaussian distribution).

*We thank the reviewer for bringing up the abbreviation inconsistency of 10x Genomics Chromium. In the revised manuscript, we provide the full name of 10x Genomics Chromium at its first appearance and abbreviate it as 10xG onwards. We also summarize all the abbreviations used in the manuscript in Additional File 1 Table S9.*

4.	Software dependencies. ‘simATAC’ depends on Snaptools which is further dependent on Python 2.X and R version 3. However, the Python 2.X has been obsolete after Dec of 2019, the current active R is version 4, and there are quite a lot of changes between Python2 and Python3, between R 3.X and R 4.X. Please make sure this new tool can work on the currently active Python and R environment.

*We thank the reviewer for pointing out the concern of software dependencies. We have tested the installation and functionality of simATAC in R versions 3.X and 4.X on Mac OS and Linux systems. simATAC can be installed and run successfully for the aforementioned R versions. Our team is committed to maintaining the compatibility of simATAC.*

*One of the main functionalities and advantage of simATAC is that it can readily simulate scATAC-seq data given user-specified parameters such as library sizes, bin means, and sparsity, without any real data. However, if the users wish to simulate scATAC-seq data that resemble real data, simATAC offers the functionality to generate synthetic bin-by-cell matrices with real scATAC-seq bin-by-cell matrices as input. In this work, Snaptools is used to preprocess raw FASTQ or BAM files to convert real scATAC-seq data to bin-by-cell matrices that can be taken as the input to simATAC. This conversion from raw files to bin-by-cell matrices can also be accomplished using other software, or customized pipelines. Regardless, Snaptools (https://github.com/r3fang/SnapTools) is compatible with Python 2 and Python 3. We have also tested the installation and functionality of Snaptools on Python 3.7.0.*

5.	Please discuss the potential usage of the simulated single-cell ATAC-seq data while evaluating computational tools. Authors showed that simATAC can mimic real data. However, in reality, why should users use these simulated data to evaluate their tools instead of simply testing their tools on real data which the simulation is based on?

*We thank the reviewer for this comment. We included the following discussion on the potential usage of the simATAC simulator, which is also included in Discussion and conclusion section of the manuscript.*

*To our best knowledge, simATAC is the first scATAC-seq simulator that directly simulates bin-by-cell count matrices that are reproducible and closely resemble real data. Though the availability of real scATAC-seq data has been increasing, real scATAC-seq data with annotated labels (“ground truth”) remains lacking. simATAC offers users the convenience to generate scATAC-seq data with known cell types and desirable number of single cells, yet closely resemble the real data. Further, simATAC provides the users the flexibility to adjust the library size, bin sparsity and noise level of the simulated scATAC-seq data. We envision that simATAC empowers users to develop scATAC-seq analytical tools effectively and reproducibly.*

Minor comments:

6.	Please double-check if the description at the end of the Result section "Bin by cell count matrix" on the "SCESet" object is right. According to simATAC tutorial at https://github.com/bowang-lab/simATAC/blob/master/Docs/tutorial.md, it should be just called as "SCE" object.

*We thank the reviewer for pointing out this typo. simATAC uses the SingleCellExperiment class from SingleCellExperiment R package in its implementation. We have corrected this abbreviation in the revised manuscript and added to Additional File 1 Table S9.*

Response to Reviewer #2:

Response to the main concern:

[[...]]

The manuscript "simATAC: A Single-cell ATAC-seq Simulation Framework" by authors Ghaziani et al., presents a new tool to create synthetic single cell ATAC-seq (scATAC-seq) data derived from experimentally generated scATAC-seq characteristics. Unlike other tools, simATAC segments the genome into equally-sized bins before simulating data based on three parameters: library size, sparsity, and average number of reads per bin. simATAC also contains a feature to control the amount of simulated noise in the synthetic data set. The authors describe simATAC as a means to generate synthetic scATAC-seq data for software benchmarking, but it is likely to be useful in other instances where simulated data is required.

One of the biggest advantages of simATAC is that it is tested against data generated from three of the most common scATAC-seq technologies. Technology significantly influences the quality and characteristics of scATAC-seq data, but simATAC simulates data that is consistently similar to real data regardless of the platform. The authors also demonstrate how adjusting the noise parameter influences the clustering analyses. Given the impact of the signal-to-noise ratio on peak detection, this is an important factor when benchmarking peak-calling software. And perhaps most important from a user's perspective, the simATAC software is well documented and its tutorial is thorough.

There are a few points that need to be addressed in the manuscript.

Major comments:

1. Firstly, in Figure 3 and several supplemental figures, the cell sparsity of simulated data is consistently lower than that of real data. It would be helpful to simATAC users if the authors addressed this.

*We thank the reviewer for the suggestion to discuss the discrepancy in the cell sparsity between the simulated and the real datasets. In the revised manuscript, we added a new input parameter to the simATAC simulation pipeline, which offers users the flexibility to adjust the sparsity as desired. The sparsity adjustment factor, , is set to 1 by default. When is set to be smaller than 1, the simulated scATAC-seq data tend to be more sparse, and vice versa.*

*In Additional File 1 Figure S7, we demonstrate the distribution of library size, cell sparsity, and bin sparsity parameters for = {0.8, 0.9, 1, 1.1, 1.2} of the simulated scATAC-seq data based on the LMPP cell group of the Buenrostro2018 dataset. While slight changes in the sparsity factor don't significantly shift the distribution of parameters, it enables users to generate synthetic data with varying sparsity. Based on our analysis, values of 0.8, 0.9, and 1 are the best values in terms of resembling the bin sparsity of input data. The Q-Q plot representation of real and simulated bin sparsity of 12 sample cell groups are provided for sparsity adjustment factor of 0.8, 0.9, and 1 (Additional File Figure S4-S6).*

*We also evaluated the downstream clustering performance of the simulated scATAC-seq data by simATAC varying = {0.8, 0.9, 1, 1.1, 1.2}, and the results are summarized in Additional File 1 Table S7. The clustering results are generally consistent at different values of , and we hence set the default value of to 1 as a suggestive value to the users.*

2.	In these same figures the authors plot bin sparsity of real and simulated data. Due to the nature of this metric, the majority of data points cluster between 0.75 and 1.0, making it difficult to determine if there are differences in median and quartile distributions as is observed with the cell sparsity plots. It would be useful if the authors plotted these differently to increase the resolution in the bin sparsity plots.

*We thank the reviewer for this comment on the visualization of the bin sparsity comparison. In the revised manuscript, we demonstrate the similarity of bin sparsity between the real and the simulated datasets using Q-Q plots (Figure 3 and Additional File 1 Figure S2 and S4). We also demonstrate the bin sparsity similarity varying the sparsity parameter = 0.8, 0.9, and 1 in Additional File 1 Figure S4-6. These figures, at higher resolutions, confirm that the bin sparsity of the simulated dataset is similar to that of the real dataset.*

3.	Table 3 compares the similarity between real and synthetic data using three metrics (MAD, MAE, RMSE), and presents these data as an average of all cell groups. Since scATAC-seq data varies significantly based on individual cell type, it would be useful to describe the range of variation across the tested cell groups.

*We thank the reviewer for this comment. We included the standard deviations of the MAD, MAE, RMSE metrics across 20 simulation runs in the revised Table 3. The small values of these standard deviations suggest the similarity of the simulated data to the real data across simulation runs and various cell groups.*

4.	Finally, Table 4 shows peak assignment similarities in simulated and real data. However, the authors only use the peak caller SnapATAC. Like simATAC, SnapATAC segments the genome into 5kb bins. To robustly demonstrate the similarity between simulated and real data sets the authors should also apply a non-segmenting peak caller. Because of the variety in peak calling software, it would be useful to compare real and simulated data using different approaches.

*We thank the reviewer for the comments. In the revised manuscript, we conducted new analyses and compared the overlapping peaks of simulated data by simATAC with those extracted using MACS2 and Genrich, two commonly used non-segmenting peak callers for scATAC-seq data. From the simulated bin-by-cell matrices by simATAC, we selected the top 30,000 bins with the highest bin means and defined them as the peaks. We then aligned the simulated matrices by simATAC and the peak-by-cell matrices extracted using MACS2 and Genrich, and compared the percentage of overlapped peaks using the three benchmark datasets as input. Additional File 1 Table S3 summarizes the average of peaks having overlap relative to the average of peak lists’ lengths, which shows that the bin-by-cell matrices simulated by simATAC is able to preserve most of the accessibility information.*

*A more conventional approach to compare segmenting and non-segmenting peaks is through down-streaming analysis. Thus, in addition to comparing overlapped peaks, we also compared the downstream analysis performance of the peak-by-cell matrices extracted using MACS2 and Genrich with the peak-by-cell matrices extracted from the simulated data by simATAC. We used Seurat to perform clustering analysis on the aforementioned matrices on the three benchmarking datasets, and the clustering results are provided in Additional File 1 Table S4, Figure S8. Note that we used Seurat instead of SnapATAC because SnapATAC is designed for analyzing bin-by-cell matrices. The solid lines in Additional File 1 Figure S8 represents the clustering metrics (NMI, AMI, ARI) of the simulated peak-by-cell matrices, varying the number of peak bins (30000, 40000, 50000 and 60000) and Gaussian noise levels (mean: 0, std: 0; mean: -0.3, std: 0.3; mean: -0.4, std: 0.4; mean: -0.5, std: 0.5). The dotted lines represent the clustering metrics of the extracted peak-by-cell from real bin-by-cell matrices. Each reported metric in Additional File 1 Figure S8 and Table S4 is based on the average of 20 simulation runs, which demonstrates the resemblance of the simulated peak-by-cell matrices to the real data as well as its robustness.*

*The new downstream analysis results as well as the peak overlapped percentage prove that the extracted peak-by-cell matrices based on simATAC simulated data maintain the cell-specific accessibility information of the real input data. As the main scope of the proposed simATAC simulator is on generating bin-by-cell matrices, we move all the analyses based on the peak-by-cell matrices extracted from the bin-by-cell matrices to the Additional File 1. The original Table 4 on peak region overlapping is now Additional File 1 Table S3.*

**Second round of review**

**Reviewer 1**

Thanks for addressing my previous questions! I don't have further concerns.

**Reviewer 2**

The manuscript “simATAC: A single-cell ATAC-seq Simulation Framework” by authors Ghaziani et al. has presented a tool that generates synthetic single cell ATAC-seq data based on a combination of experimentally derived scATAC-seq characteristics and user-specified parameters. In this re-submission, the authors have adequately addressed my previous comments
